# Prognostic and diagnostic utility of heart rate variability to predict and understand change in cancer and chemotherapy related fatigue, pain, and neuropathic symptoms: a systematic review

**DOI:** 10.1007/s00520-025-10164-x

**Published:** 2025-11-11

**Authors:** Jessica Bolanos, Layal Hneiny, Juan Gonzalez, Maximilian O’Malley, Marlon L. Wong

**Affiliations:** 1 APReCIAT Laboratory, Department of Physical Therapy, Miller School of Medicine, University of Miami, Miami, FL USA; 2 Miller School of Medicine, Louis Calder Memorial Library, University of Miami, Miami, FL USA

**Keywords:** Heart rate variability, Cancer, Fatigue, Pain, Neuropathy

## Abstract

**Purpose:**

Advances in early cancer detection and treatment have significantly improved survival rates, resulting in over 18.1 million cancer survivors in the USA. Many of these survivors experience chronic pain, fatigue, and neuropathic symptoms related to cancer or its treatments. Emerging evidence suggests that autonomic nervous system dysfunction plays a crucial role in these symptoms. Heart rate variability (HRV), a measure of autonomic function, has shown potential in predicting the onset and progression of these cancer-related symptoms. This systematic review aimed to assess the association of HRV with pain, fatigue, and neuropathy in cancer patients and survivors.

**Methods:**

A comprehensive search was conducted across multiple databases, yielding 23 studies that met inclusion criteria. These studies varied in cancer types, stages, and HRV measurement methods.

**Results:**

Most studies focused on breast cancer and reported a predominant female population. Fatigue was the most studied symptom (*n* = 15), followed by pain (*n* = 7), and only one study assessed neuropathic symptoms. HRV measures included both time and frequency domain variables, with significant variability in measurement duration and control for confounding factors.

**Conclusion:**

Findings suggest that decreased HRV is associated with increased fatigue and pain, providing potential support for a bidirectional relationship between autonomic dysfunction and these symptoms. However, the heterogeneity in HRV measurement methods and the high risk of bias in many studies highlight the need for high-quality prospective and interventional studies with standardized HRV protocols in cancer research.

**Supplementary information:**

The online version contains supplementary material available at 10.1007/s00520-025-10164-x.

## Introduction

**Fig. 1:**
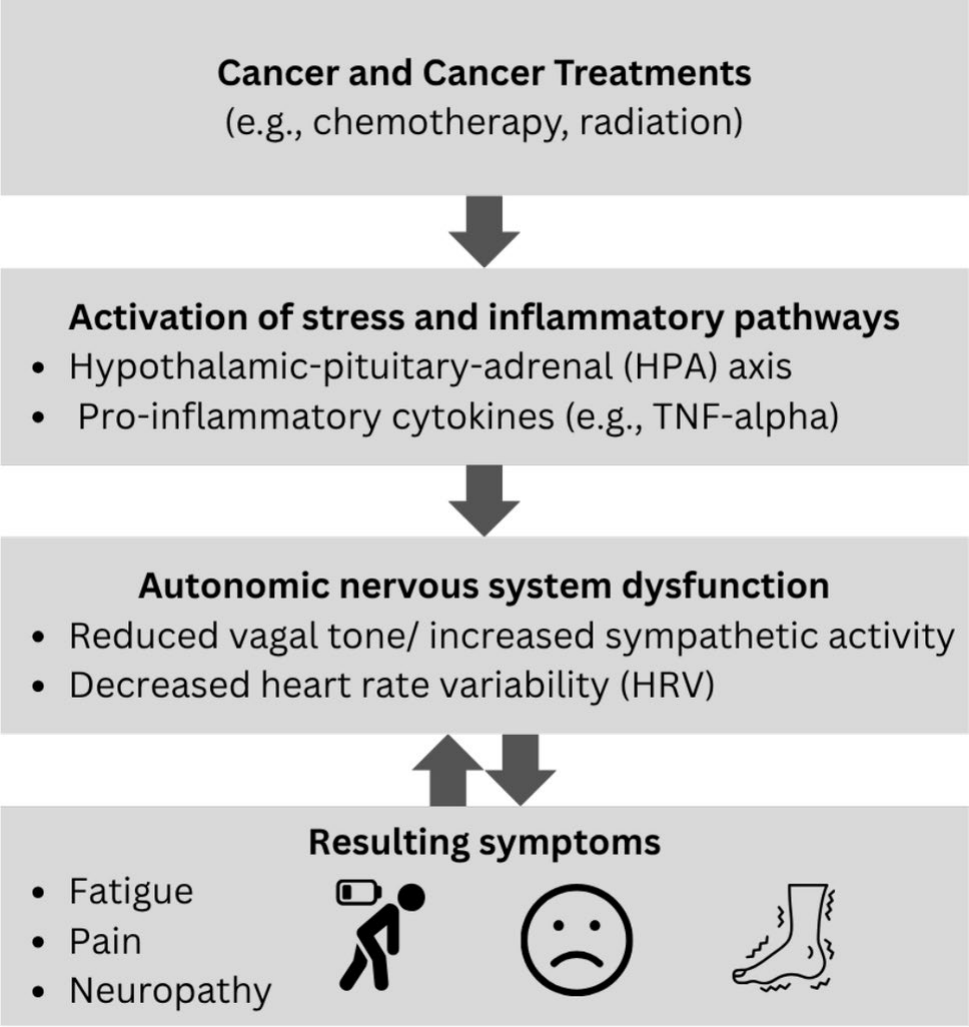
Conceptual model of heart rate variability and symptom burden

Advances in early detection and treatment have significantly improved the 5-year cancer-specific survival rates [1]. As a result, there are currently over 18.1 million cancer survivors in the USA [1], many of whom suffer from chronic pain [2], fatigue [3], or neuropathic symptoms (i.e., paresthesia and dysesthesia) associated with cancer or cancer treatments [4, 5]. There is growing evidence that dysfunction of the autonomic nervous system plays a key role in the development and maintenance of these comorbid symptoms [6–8]. The autonomic nervous system is an important regulator of stress responses, and it exhibits maladaptive functional changes in chemotherapy-induced peripheral neuropathy (CIPN) and in other chronic pain conditions [8, 9]. The vagus nerve is the primary nerve of the parasympathetic system, and it is known to modulate pain through three pathways: (1) by maintaining autonomic and hypothalamic–pituitary–adrenal axis balance thereby reducing allostatic load [10], (2) via the cholinergic anti-inflammatory pathway in which action potentials in the vagus nerve inhibit the production of TNF and other inflammatory cytokines [11], and (3) by modulating the function of brain networks involving the cingulate cortex and insula which are involved in pain processing [12]. Emerging evidence suggests that dysfunction of these same pathways may also contribute to cancer-related fatigue and neuropathy [8, 9, 13] (Fig. 1).

Measures of heart rate variability (HRV) are commonly used as surrogate measures of autonomic function/vagal tone, and recent studies suggest that changes in HRV may predict the onset of comorbid pain, fatigue, and neuropathy symptoms in cancer patients. In fact, one study found that decreases in HRV preceded the onset of CIPN symptoms in people receiving oxaliplatin for gastrointestinal cancer [8], and another study found that decreases in HRV were correlated to sensory disturbance in people with CIPN [9].

HRV is a complex measure involving the fluctuation in the time intervals between heartbeats. These changes can influence regulatory outputs like blood pressure, heart and vascular tone, etc., which aids adaptation to both physical and psychological stressors [14]. This physiological link has allowed HRV to be used as an autonomic marker for cardiovascular risk stratification [15]. Decreased HRV has also been observed in other chronic pain populations, and it has been proposed that there is a bidirectional relationship between chronic pain and autonomic dysfunction [16]. Therefore, HRV is a promising measurement tool for improving the phenotyping and measurement of cancer-related symptoms, and it is potentially a useful treatment target.

The purpose of this systematic review study was to assess the association of HRV with comorbid symptoms (i.e., pain, fatigue, and neuropathy) in cancer patients and survivors. However, the use and interpretation of HRV lack standardization in research. Thus, this review also describes the methods used for HRV measurement in cancer research and discusses how these methods may influence findings.

## Materials and methods

A systematic review was conducted following the Methodological Expectations of Cochrane Intervention Reviews Manual [17], and the results were reported according to the Preferred Reporting Items for Systematic Reviews and Meta-analyses (PRISMA) [18]. The review methods were established before developing the review, and the study protocol was registered at the International Prospective Register of Systematic Reviews under CRD42023418747.

### Search strategy

The study inclusion criteria consisted of (1) studies including cancer patients or survivors, (2) longitudinal cohort studies and randomized control trials (RCTs) that include at least one HRV measure timepoint and must include an objective measure of change for at least one of the symptoms listed (pain, fatigue, and/or neuropathy), or (3) cross-sectional studies that compare HRV between groups of cancer survivors with and without one of the symptoms of interest and allow for calculation of effect sizes. A search strategy was built by the clinical research librarian (LH) from Louis Calder Memorial Library together with the principal investigator (MW). A peer review of the search strategy was conducted by another medical librarian (DG) who provided input using the Peer Review for Electronic Search Strategies tool [19].

Firstly, the search strategy was developed in the database Ovid using both the Medical Subject Headings for the concepts of Heart Rate Variability, Pain and Fatigue, and cancer and chemotherapy and their alternate keywords. In addition to Ovid MEDLINE(R) ALL, the following databases were searched: Cochrane Central Register of Controlled Trials (CENTRAL) (Cochrane Library, Wiley); Embase (Elsevier, Embase.com); Cumulative Index to Nursing and Allied Health Literature (CINAHL) Plus (Ebsco); the Web of Science platform (Clarivate: Science Citation Index Expanded, Social Sciences Citation Index, Arts & Humanities Citation Index, Conference Proceedings Citation Index-Science, Conference Proceedings Citation Index-Social Science & Humanities, Emerging Sources Citation Index), Scopus (Elsevier), PsycINFO, ProQuest Databases (ABI/INFORM Collection, Academic Video Online, AFI Catalog, American Periodicals, ASFA: Aquatic Sciences and Fisheries Abstracts, Black Studies Center, Business Market Research Collection, Coronavirus Research Database, Digital National Security Archive, Dissertations & Theses @ University of Miami, Early Modern Books, Ebook Central, ERIC, Ethnic NewsWatch, GenderWatch, Global Newsstream, Healthcare Administration Database, Music Periodicals Database, ProQuest Civil War Era, ProQuest Dissertations & Theses Global, ProQuest Historical Newspapers: Chicago Defender, ProQuest Historical Newspapers: New York Amsterdam News, ProQuest Historical Newspapers: New York Tribune, ProQuest Historical Newspapers: Philadelphia Tribune, ProQuest Historical Newspapers: The Baltimore Afro-American, ProQuest Historical Newspapers: The New York Times with Index, PTSDpubs, Publicly Available Content Database, Research Library, SciTech Premium Collection, Sociological Abstracts, Teatro Español del Siglo de Oro, Worldwide Political Science Abstracts) limited to Dissertations & Theses, and APA PsycInfo (Ebsco).

The Medline search strategy was adapted for the other databases each according to its syntax taking into consideration the use of different controlled vocabulary such as EMTREE terms for Embase, Medical Subject Headings for Cochrane Library, and CINAHL Subject Headings for CINAHL Plus, in addition to revising the truncation, adjacency, and search fields. No application for language, date, or other limitations was applied for the initial query; however, only manuscripts in English were selected for inclusion. All databases were initially searched from November 8 to 10, 2023, and the search was repeated from February 25 to 27, 2025. The full search strategy is available in Appendix 1.

#### Study selection, data extraction, and data management

The search results were exported into Covidence Web-based software [20] directly or into EndNote 20 [21] then imported to Covidence. In Covidence, the duplicates of overlapping results were removed, then the remaining results were first screened for title-abstract and then screened for full-text [20]. Each step was conducted independently by two reviewers (JB and JG for abstract review; JB and MO for full-text), and conflicts were resolved by consensus with the primary investigator (MW). Moderate agreement was observed between raters (*k* = 0.531 for JB and JG; *k* = 0.574 for JB and MO) [22]. Percent agreement (number agreed/total number of articles) was calculated at 99% (6591/6677 for JB and JG) and 98% (755/768 for JB and MO).

An Excel spreadsheet was developed and piloted for data extraction. Then, two independent reviewers (JB and MO) extracted data from the full-text manuscripts. Missing data was requested from the study authors via email. Seven authors were contacted in November 2024 via email requesting clarification of measures or raw datasets for symptoms of interest and heart rate variability measures. Two authors responded with clarifications, two contacts were listed as undeliverable, and three authors did not respond.

#### Risk of bias assessment and synthesis of findings

Our intention was to include all longitudinal studies with repeated measures of HRV and symptoms, which would likely encompass a variety of study designs. Thus, the Mixed Methods Appraisal Tool (MMAT) was chosen for risk of bias assessment due to its applicability to a wide range of study types [23]. Each study was assessed by two independent reviewers (JG and MO) for risk of bias, and disagreements between reviewers were resolved by deliberation until a consensus was reached (Appendix 2).

The body of evidence was assessed based on the Cochrane GRADE classification (high, moderate, low, and very low) [24], and an Excel spreadsheet was used to record the data. Four investigators (JB, JG, MO, and MW) deliberated until consensus was achieved for the Cochrane GRADE classification.

## Results

### Study characteristics

**Fig. 2:**
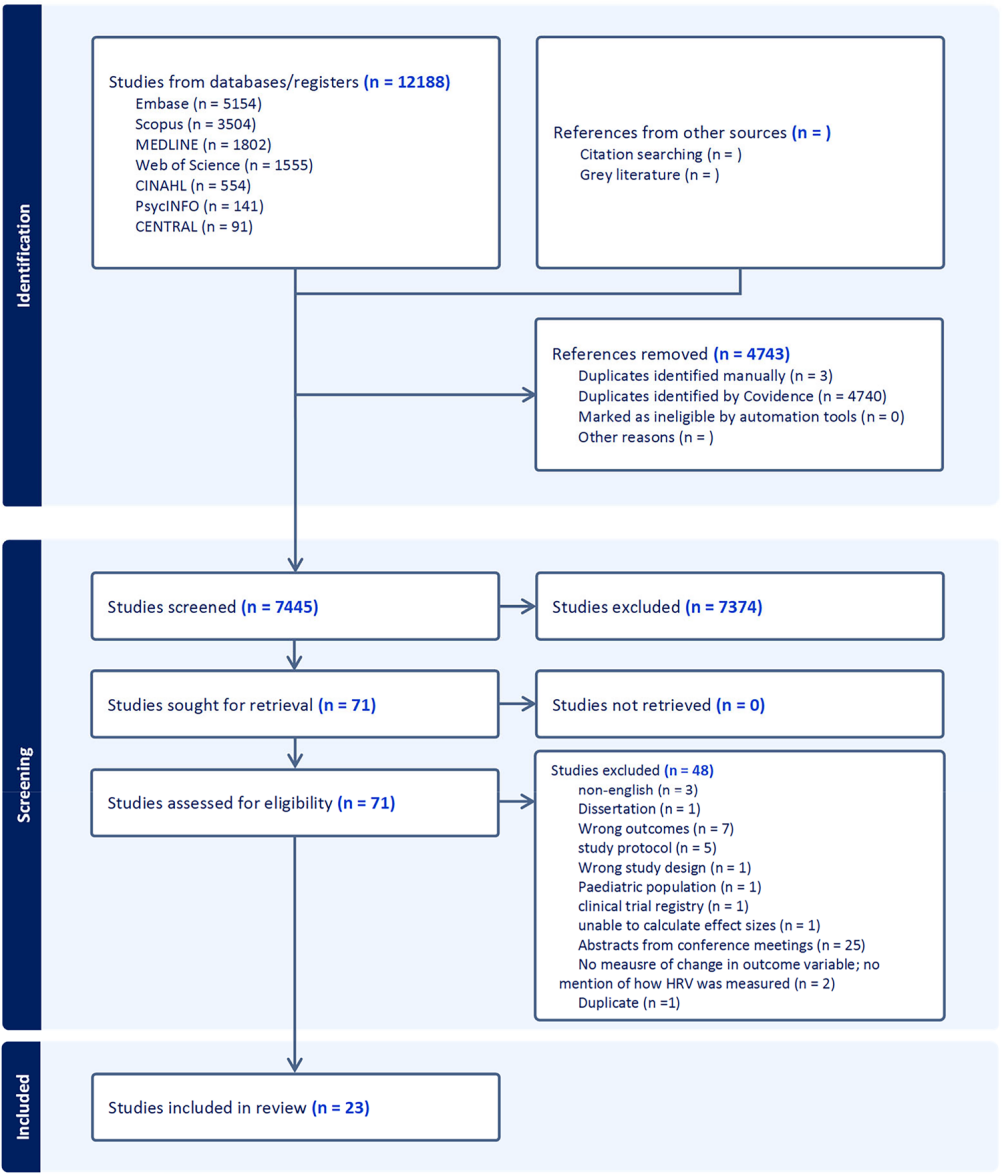
PRISMA diagram

A total of 7445 titles and abstracts were screened by two reviewers. Seventy-one full-text articles were assessed with 23 articles ultimately selected for inclusion (Fig. 2). Publication dates ranged from 2009 to 2025, and the studies covered a wide range of cancer types (Table 1). Nine of the 23 studies focused solely on people with breast cancer [25–33]. Of the 23 studies, 9 [29, 31, 33–39] did not report cancer staging information. In the 15 studies that reported staging, participants ranged from early stage (0, I, II) to late stage (III, IV) with most participants in stage I.

**Table 1:** Study characteristics

First author (year)	Country	Aim of study	Design	Participants recruited	Heart rate variability measurement details	Key relevant findings
Tsai (2021)	Taiwan	To assess what factors may influence the outcome of cancer related fatigue among female breast cancer patients undergoing chemotherapy	Cohort study	109 patients undergoing cancer treatment	15-min recording, no positioning information given; ECG device; frequency domain as primary	Anxiety, depression, and symptom distress contributed to cancer related fatigue with symptom distress, anxiety, and sleep deprivation consistently high among those receiving chemotherapy. No predictive relationship was found between LF/HF ratio and fatigue levels
Riesenberg (2009)	Germany	To assess if intensive supervised aerobic exercise is efficacious in improving HRV, quality of life, and functional status for patients with lung cancer over a 28-day period in inpatient rehab?	Cohort study	45 patients undergoing cancer treatment	12-min recording laying down; PPG device; time domain as primary	Exercise capacity and functional status (quality of life, fatigue) improved during rehabilitation. RMSSD increased post intervention
Aoki (2023)	Japan	To assess if HRV would be useful in evaluating fatigue and quality of life of women with breast cancer undergoing radiotherapy	Cohort study	75 patients undergoing cancer treatment	2-min recording, sitting; PPG device; frequency domain as primary	Cognitive and physical fatigue were highest at baseline and decreased by midpoint of treatment. Similarly, a decrease in LF/HF ratio was seen by midpoint of treatment
Fagundes (2011)	USA	To assess if fatigue level is related to HRV or epinepherine at baseline and during/after a social stressor in breast cancer survivors	Cohort study	109 cancer survivors	Continuous recording, sitting; PPG device; time domain as primary	Higher fatigue was associated with decreased RMSSD values pre and post stressor
Yesil (2018)	Turkey	To assess whether neuropathic pain is associated with changes in cardiac sympathovagal activity in patients with breast cancer	Cohort study	70 patients undergoing cancer treatment	24-h recording, no positioning information given; ECG device; time domain as primary	SDNN was lower and LF/HF ratio was higher in patients with neuropathic pain than in those without
Fournié (2021)	France	To evaluate the feasibility of a 12-week program combining heart rate variability biofeedback and physical exercise in hematologic patients	Cohort study	20 patients undergoing cancer treatment	5-min recording, seated with knees at 90°, feet flat on the floor, hands on thighs, palms facing upward, and eyes closed; PPG device; time domain as primary	No changes in fatigue outcome measures noted despite a trend towards decreasing general/physical fatigue. SDNN and RMSSD did not change over time, but increased LF and HRV coherence ratio were noted
Fujisawa (2024)	Japan	To objectively verify the effectiveness of animal-assisted therapy as a complementary based on its effects on those with terminal cancer	Cross sectional study	36 patients in hospice care; 9 healthy participants	No recording or positioning information given; ECG device; frequency domain as primary	Mood scales improved with intervention but there was no difference in average pain values pre/post intervention. LF/HF ratio in the patient group significantly decreased during and immediately after the intervention; no change was noted with the healthy group
Guimond (2018)	Canada	To assess if measures of emotional regulation are associated with measures of psychological symptoms in patients with breast cancer receiving radiotherapy	Cross-sectional	81 patients undergoing cancer treatment	5-min recording, no positioning information given; PPG device; time domain as primary	Low emotional regulation was marginally associated with increased depression and fatigue between timepoints. HF was not associated with symptom change
Fernandez-Lao (2011)	Spain	To investigate the immediate effects of myofascial release on HRV and mood state and to assess the attitude towards massage in breast cancer survivors suffering cancer-related fatigue	Crossover randomized control trial	20 cancer survivors	5 min ECG, no positioning information given; ECG device; time domain as primary	Massage leads to an immediate increase in SDNN, RMSSD and HF and an improvement in mood. The impact of massage on cancer-related fatigue is modulated by the attitude of the patient towards massage; however, no interaction was reported between belief and SDNN, RMSSD, or HF values
Chai (2024)	China	To assess the impact of Managing Cancer and Living Meaningfully (CALM) intervention compared to usual care in cancer related fatigue, quality of life, and HRV in patients with lung cancer	Non-blinded randomized control trial	153 patients undergoing cancer treatment	10 min recording; supine position; ECG device; time domain as primary	As the intervention progressed, significant reduction in cancer related fatigue was noted. Increases in SDNN and HF compared to baseline were seen in the CALM group; SDNN and HF significantly decreased in the usual care group
Burch (2020)	USA	To assess if heart rate variability biofeedback treatment among cancer survivors can increase HRV coherence and reduce symptoms of pain, stress, fatigue, depression, distress, PTSD, and insomnia relative to control patients in 6 weeks	Pilot randomized control trial	38 cancer survivors	15-min recording, no positioning information mentioned; ECG device; HRV coherence ratio reported as primary	Relative to controls, the intervention group experienced trends toward improvements in stress, distress, fatigue, PTSD, and depression. HRV coherence ratio increased following biofeedback training in the intervention group while no change or decrease in coherence ratio occurred with control group
Masel (2016)	Austria	To assess if HRV a valid representation for subjective pain for patients in palliative care with cancer break through pain	Pilot study	10 patients undergoing cancer treatment	24-h recording, no positioning data given; ECG device; frequency domain as primary	Pain reduction was seen post opioid administration. Log LF/HF ratio decreased in patients with > 2 point reduction on the NRS
Murofushi (2023)	Japan	To assess if HRV can provide an objective measure for bone metastasis pain during a period of radiotherapy	Pilot study	11 patients undergoing cancer treatment	24-h recording, no positioning information given; PPG device; frequency domain as primary	For all samples, the NRS score showed no significant relationship with mean LF/HF ratio and HF. In patients with an NRS ≥ 4, the NRS score was significantly associated with LF/HF ratio but not HF
Chuang (2010)	Taiwan	To assess if music therapy is effective in lowering anxiety, improving mood, and improving HRV when applied for a 2-h session one time intervention in cancer survivors	Quasi-experimental	23 cancer survivors	5-min recording, no positioning information given; ECG device; frequency domain as primary	Fatigue levels were lower after music therapy. Increase in HF power and decrease in LF/HF ratio and LF power were observed post intervention
Lee (2022)	Taiwan	To assess if there is a difference in HRV measures and quality of life measures when given either Qigong, mindfulness intervention or no intervention in cancer survivors at least 1 month out of treatment	Quasi-experimental	125 cancer survivors	5-min recording, sitting; PPG device; time domain as primary	Both Qigong and mindfulness interventions had short-term effects in improving overall physical and mental health, and quality of life. Increase in SDNN and total power were noted in the short term, but decreased LF was reported in the long term
Niederer (2013)	Germany	To assess if exercise over a 16-week period improves HRV measures and cancer related fatigue in both cancer survivors and patients undergoing cancer treatment	Quasi-experimental	45 cancer survivors and those undergoing treatment	5-min recordings, supine; PPG device; frequency domain as primary	Exercise programing improves quality of life and increases total power values. An increase in HF and decrease in LF was also observed post intervention
Lee (2018)	Taiwan	To assess the effects of 12 weeks of Qigong exercise or stress management training on fatigue, fear of recurrence, quality of life, and heart rate variability in people who completed cancer treatment at least 1 month prior	Randomized control trial	99 cancer survivors	5-min recording seated or lying prone; PPG device; time domain as primary	After 12 weeks of both Qigong and stress management training, fatigue was reduced, and increases in SDNN, TP, and HF were observed
Cheng (2019)	Taiwan	To assess if 12 weeks of mindfulness or Qigong training change outcomes for fatigue and HRV in cancer survivors	Randomized control trial	66 cancer survivors	5-min recording; no positioning information given; PPG device; time domain as primary	Reduced cancer-related fatigue was present in both the mindfulness group and the Qigong group. SDNN and TP values also increased from baseline
Chen (2019)	Taiwan	To assess the effect of a single music therapy intervention on multiple mood categories and HRV for women actively being treated for breast cancer	Randomized control trial	100 patients undergoing cancer treatment	5-min recording, no positioning information given; ECG device; frequency domain as primary	Women who completed single session music intervention reduced symptoms post-intervention compared to control group. Higher LF/HF ratio reported at baseline, but no post-intervention data was given
Werthmann (2025)	Germany	To investigate the effect of rhythmic embrocation, a massage technique, on post operative stress levels in post operative patients with colorectal cancer compared to empathic conversation	Randomized control trial	68 patients undergoing cancer treatment	Recorded overnight; no positioning information given; ECG device; time domain as primary	Stress decreased more in the professional rhythmic embrocation group. SDNN improved the most in the professional rhythmic embrocation group, but this was not statistically significant
Hohneck (2025)	Germany	To assess the differential effects of a receptive music therapy with sound interventions tuned to 432 Hz or 443 Hz on cardiovascular parameters and psychological outcomes in cancer	Randomized crossover trial	45 patients undergoing cancer treatment	No recording information given; supine positioning; oscillometric device; time domain as primary	Reduction in fatigue, anxiety, and stress was seen for both groups. HRV (as measured by RMSSD) was increased only in the 432 Hz group
Uchida (2017)	Japan	To assess if HRV is predictive of PACU emergent pain within a 12 h hour window in post operative breast cancer surgery patients	Retrospective study	20 patients undergoing cancer treatment	No positioning or recording time information given; PPG device; frequency domain as primary	There was no significant difference in pain intensity between the two groups immediately after admission to PACU. Patients in the intervention group required no analgesic agents for at least 12 h after surgery. Increased HF and decreased LF/HF ratio values were associated with adequate pain intervention
Uchida (2017)	Japan	To assess if low dose Remifentanil effects post operative pain and HRV in breast cancer surgery patients	Retrospective study	20 patients undergoing cancer treatment	No positioning or recording time information given; PPG device; frequency domain as primary	After Remifentanil administration, the value of the NRS decreased. There were no significant differences in HF and LF/HF ratio was found between groups

*PPG*,  photoplethysmogram; *SDNN*, standard deviation of the normal-to-normal interval; *TP*, total power; *HF*, high frequency power; *PTSD*, post-traumatic stress disorder; *HRV*,  heart rate variability; *ECG*, electrocardiogram; *LF/HF* ratio, low frequency/high frequency ratio; *RMSSD*,  square root of mean squared differences of successive normal-to-normal intervals; *NRS*, numeric rating scale; *LF*, low frequency; *PACU*, post-anesthesia care unit

A majority of the participants across studies were middle-aged (ranging from 48 to 65 years). All but four of the studies [37–40] reported a predominant female population, with ten having an all-female sample [25–34]. One study did not provide gender information [41]. Studies took place in Taiwan (*N* = 6)[25, 27, 34, 36, 42, 43], Japan (*N* = 5) [28, 29, 33, 39, 44], the USA (*N* = 2)[30, 45], Germany (*N* = 4)[37, 40, 41, 46], Canada (*N* = 1)[32], France (*N* = 1)[38], Turkey (*N* = 1)[31], Spain (*N* = 1)[26], Austria (*N* = 1)[35], and China (*N* = 1) [47]. Only two studies provided detailed data on race or ethnicity, both of which took place in the USA [30, 45].

Fatigue was the most common symptom discussed (*N* = 15)[25–28, 30, 32, 34, 36–38, 41–43, 47] followed by pain (*N* = 8)[29, 33, 35, 39, 40, 44, 46]. Although one study assessed neuropathic pain [31], no studies were found on non-painful neuropathic symptoms, and only one study assessed multiple symptoms (i.e., pain and fatigue) [45]. Four of the included studies focused on the psychological effects of cancer (mood, anxiety, depression) as a primary measure with fatigue as a secondary measure of interest [25, 26, 34, 43].

#### Heart rate variability measures

Most studies measured HRV using electrocardiogram (ECG) (*N* = 9) or photoplethysmography (PPG) (*N* = 13) equipment in the form of wrist watches, handheld or chest strap tools for HRV measurement. One study utilized an oscillometric device [46]. Eleven studies conducted HRV measurements for 5 min or less [26–28, 32, 34, 36–38, 42, 43, 47], three reported recording times between 12 and 15 min [25, 41, 45], and four reported continuous measurement for 24 h [30, 31, 35, 39]. Five studies did not report on the duration of the recording [29, 33, 40, 44, 46]. Studies varied in participant positioning during HRV recording and in the level of control for confounding factors (i.e., caffeine, physical activity), with only 10 of 23 studies failing to include this information in the manuscript [25, 29, 33, 35, 36, 39, 43, 44, 47].

#### Heart rate variability and fatigue

Time domain measures (SDNN, RMSSD) were reported as primary outcomes in six studies, with five showing a consistent association between increased HRV measures and reduced fatigue. Frequency domain findings were more variable with some studies reporting decreased LF/HF ratios or increased total power with reduced fatigue, while others found no significant changes.

Four additional studies assessed fatigue as a secondary outcome, primarily in the context of psychological interventions to address anxiety, depression, or fear of cancer recurrence. These studies also demonstrated improvements in HRV (increased SDNN, RMSSD, HF; decreased LF/HF ratio) alongside reduced fatigue, except for one study with no significant difference.

**Fig. 3:**
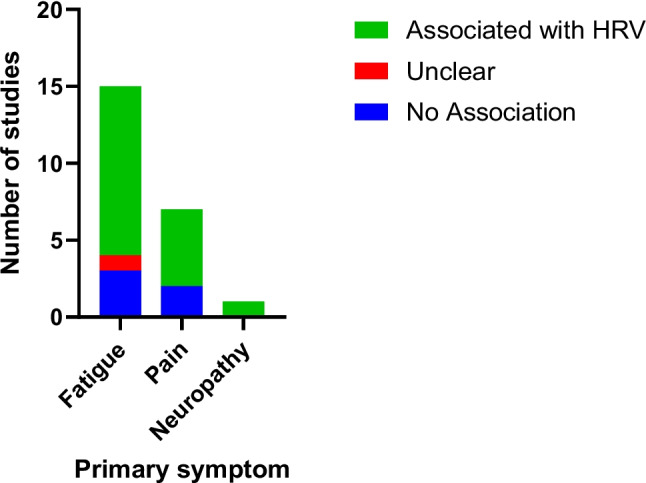
Summary of heart rate variability (HRV) findings

Overall, 11 of 15 studies examining fatigue found a positive connection between improved HRV and reduced fatigue (Fig. 3), particularly in time domain metrics. However, the GRADE confidence rating for this evidence was low, due to methodological limitations including incomplete data, the lack of confounder control, and the absence of blinding (Table 2).

**Table 2:** Summary of Mixed Methods Appraisal Tool (MMAT) findings

Symptom domain	Number of studies	GRADE confidence	MMAT risk of bias summary
Fatigue	15	Low	Incomplete outcome data in five studies, lack of confounder control in six, no blinding in three
Pain	7	Very low	High risk of bias due to incomplete reporting and lack of confounder control; small sample sizes
Neuropathy	1	Not rated	Single study; insufficient evidence for GRADE rating

*GRADE*, The Grading of Recommendations Assessment Development and Evaluation. GRADE assessments of certainty are determined through consideration of five domains: (1) risk of bias, (2) inconsistency, (3) indirectness, (4) imprecision, and (5) publication bias. Low: Our confidence in the effect estimate is limited, and the true effect may be substantially different. Very low:  We have very little confidence in the effect estimate, and the true effect is likely to be substantially different. *MMAT*, The Mixed Methods Appraisal Tool

#### Heart rate variability and pain

Seven studies focused on pain, primarily using single-item intensity scales (NRS, VAS, Wong-Baker). Sample sizes were generally small (≤ 20), and participants ranged in cancer stage of treatment from post-operative to hospice care. Only one study controlled for HRV confounders. Most studies used frequency domain measures, with three also reporting time domain metrics.

Four studies assessed acute pain (e.g., postoperative, breakthrough), with three showing that reductions in pain intensity were associated with decreased LF/HF ratios. One study reported increased SDNN post-intervention, though this finding was not statistically significant. Two studies on chronic pain (i.e., bone metastasis) found associations between LF/HF ratio changes and higher pain scores (NRS ≥ 4), but not with lower scores.

These findings suggest that moderate reductions in pain intensity (NRS change of 2–4) may correspond with improved HRV, particularly in frequency domain metrics. However, the GRADE confidence rating for pain-related HRV evidence was very low, due to a high risk of bias and imprecision (Table 2).

#### Heart rate variability and neuropathy

Only one study addressed neuropathy, using the Leeds Assessment of Neuropathic Symptoms and Signs (LANSS) scale, to differentiate neuropathic from nociceptive pain. Participants with neuropathic pain exhibited lower time-domain HRV (SDNN) and higher frequency-domain values (LF/HF ratio, TP), suggesting autonomic imbalance.

## Discussion

This study aimed to assess the relationship of HRV with comorbid pain, fatigue, and neuropathy symptoms in cancer patients and survivors. Across the 23 studies reviewed, HRV was used in various ways, including to determine the physiological burden of comorbid symptoms and as a measure of systemic regulation in response to an intervention. There was also wide heterogeneity in the measurement protocols and reporting of HRV measures.

### HRV confounders

Due to the complex and interdependent neuro-immuno-endocrine processes that govern the autonomic nervous system, HRV measurements can be influenced by a variety of variables, including age, sex, and breathing rate [14]. Further, numerous lifestyle factors including stress level, smoking, fitness level, caffeine intake, and pharmacology, can also have an influence on HRV metrics [48]. Thus, identification and control of these confounding factors is of utmost importance to decrease error in HRV interpretation. In this sample of articles, only 8 of 23 studies included this information.

Additionally, sampling time can influence the accuracy of HRV metrics. The “gold standard” for clinical assessment is 24-h continuous recording, as these recordings are shown to have greater predictive power [14]. Short-term measurements, defined as 5-min measurements, have been found to correlate poorly with the 24-h indices [14]. Of the 23 articles reviewed, 10 reported short-term measurements and only 5 utilized 24-h measurements. The variation in sampling time across studies makes it difficult to synthesize findings and contributed to our inability to perform a meta-analysis.

HRV metrics also include time, frequency, and spectral domain measures. Only time and frequency domain measures were reported in the studies reviewed. Time domain HRV indices (i.e., SDNN, RMSSD, pNN50, HRmax–HRmin) quantify the amount of variability observed during a recording time frame (< 1 min to > 24 h) [14]. SDNN is considered the “gold standard” for medical stratification of cardiovascular risk when recorded over a 24-h period [14, 15]. However, only 9 of the 23 studies reviewed reported the SDNN. In contrast, 20 studies reported frequency domain measures as primary or secondary, most commonly LF/HF ratio. Frequency domain HRV indices estimate the relative or absolute power over four frequency bands: ultra-low frequency (ULF), very-low frequency (VLF), low-frequency (LF), and high-frequency (HF) [14] The ratio of LF to HF (LF/HF ratio) was originally intended to estimate the sympathetic to parasympathetic ratio, with low LF/HF ratio symbolizing parasympathetic dominance and high LF/HF ratio translating to sympathetic dominance [14]. This association contributes to the popularity of frequency domain variables being reported over time domain. However, the accuracy of this association has been called into question due to the variable nature of sympathetic nervous system control and thus LF/HF ratio, especially short-term LF/HF ratio, has been found to vary widely based on testing conditions [14]. Kuss et al. found that frequency domain variables showed a tenfold increase in variation when compared to SDNN, which had the highest precision of all variables [49]. The observed preference for frequency domain variables over the clinical gold standard time domain variables in these studies may require further critical appraisal and highlights a lack of consensus on best practices for HRV measurement and reporting. Overall, the lack of standardization in how HRV is measured and reported further limited the potential for meta-analytic synthesis in this study. The expanding options for wearable technology may provide opportunity for widespread and easy collection of HRV data in the future; however, consensus guidelines are needed on the minimum recording duration, device types, and confounder controls.

### Symptom measure differences

Variances in how symptoms are measured could also be a contributing factor to the differences observed between studies. The seven studies on pain all used single-item measures (NRS, VAS, Wong Baker Face Scale) focusing on change in pain intensity in response to a pharmacological intervention. In contrast, the fatigue studies utilized multi-item scales and indices that assessed multiple facets of fatigue. These measurement tools included the Cancer Fatigue Scale (CFS), Fatigue Symptom Inventory (FSI), Piper Fatigue Scale, and Multidimensional Fatigue Symptom Inventory-Short Form (MFSI-SF), which have all been validated in patients with cancer and all measure physical, cognitive and emotional aspects of fatigue [50–52]. Unlike the pain studies, where a single pain construct (intensity) was the focus, the fatigue studies reported total score changes, with only one study reporting subscale changes [38].

HRV measurements in six of seven pain studies were single-session recordings, pre- and post-intervention. Furthermore, improvement in time and frequency HRV domains was found in six of seven studies following acute interventions for pain intensity, though half of those changes lacked statistical significance. In contrast, HRV measurements for fatigue were taken longitudinally (pre-intervention, midpoint of intervention, post-intervention) in seven of ten studies. Unlike the pain studies, with acute changes in HRV noted post intervention, the studies focused on fatigue reported long-term washout of acute HRV changes by post-intervention follow-up, with mixed reporting on changes in time and frequency domains but overall improvement in additional measures like quality of life [53] and the MFSI-SF [30].

### Limitations

There are several key limitations of this review study. First, we only included articles published in English, and it is likely additional pertinent research exists in other languages. Further, our search only yielded one study on neuropathy. This may have been influenced by the lack of cohesive clinical screening methods to effectively diagnose chemotherapy-induced peripheral neuropathy (CIPN) [8]. Another potential reason for this may be that CIPN is only found with neurotoxic chemotherapies such as paclitaxel, cisplatin, and oxaliplatin [54]. In this review, only 7 of 23 studies indicated some history of chemotherapy management. Additionally, the generalizability of our findings may be limited by the fact that only 2 of the 24 studies reported on race/ethnicity and 12 of 24 studies took place in Asia (Taiwan, Japan, China).

The overall confidence in the evidence was limited by methodological weaknesses (Table 2). Fatigue studies, while more numerous, often lacked blinding and adequate control for confounders. Pain studies were generally small and inconsistently reported. The single neuropathy study, though promising, was insufficient for robust conclusions. These limitations underscore the need for standardized HRV protocols and rigorous study designs in future research.

## Conclusion

HRV is growing in popularity as a measure of physiological response to high comorbid symptom burden. Fatigue and pain are common comorbidities associated with antineoplastic treatment, and the findings of this review suggest that decreased HRV is associated with fatigue and pain in cancer survivors. However, there is high heterogeneity in both HRV measurement protocols and in reporting of HRV variables, as well as a high risk of bias among existing studies. Based on the limitations identified in this review, we recommend the following priorities for future research on HRV and symptom burden in cancer survivors: adopt standardized protocols, ideally including both short-term (≥ 5 min) and 24-h recordings, to enhance reliability and comparability; systematically assess and report key confounders (e.g., age, sex, medication, caffeine, physical activity) and incorporate them into statistical analyses; standardize participant posture, activity restrictions, and environmental conditions during HRV measurement; report both time and frequency domain measures, with justification for primary metric choice based on study aims; and emphasize prospective and interventional designs to strengthen causal inference and address the limitations of cross-sectional studies. By implementing these recommendations, future studies can improve methodological rigor, facilitate data synthesis, and advance understanding of the role of autonomic function in cancer-related symptoms.

## Supplementary information

Below are the links to the electronic supplementary materials.

Supplementary file 1 (XLSX 28.6 KB)

Supplementary file 2 (PDF 64.4 KB)

## Data Availability

No datasets were generated or analysed during the current study.

## References

[1] Tonorezos E et al (2024) Prevalence of cancer survivors in the United States. J Natl Cancer Inst 116(11):1784–1790 39002121 10.1093/jnci/djae135 PMC11542986

[2] Arthur J et al (2018) Personalized pain goal as an outcome measure in routine cancer pain assessment. J Pain Symptom Manage 56(1):80–87 29526610 10.1016/j.jpainsymman.2018.03.004

[3] Prue G et al (2006) Cancer-related fatigue: a critical appraisal. Eur J Cancer 42(7):846–863 16460928 10.1016/j.ejca.2005.11.026

[4] Kerckhove N et al (2017) Long-term effects, pathophysiological mechanisms, and risk factors of chemotherapy-induced peripheral neuropathies: a comprehensive literature review. Front Pharmacol 8:86 28286483 10.3389/fphar.2017.00086 PMC5323411

[5] Seretny M et al (2014) Incidence, prevalence, and predictors of chemotherapy-induced peripheral neuropathy: a systematic review and meta-analysis. Pain 155(12):2461–2470 25261162 10.1016/j.pain.2014.09.020

[6] Stone CA et al (2012) Autonomic dysfunction in patients with advanced cancer; prevalence, clinical correlates and challenges in assessment. BMC Palliat Care 11:3 22379978 10.1186/1472-684X-11-3 PMC3314561

[7] Noor B et al (2020) Quantitative assessment of cardiovascular autonomic impairment in cancer survivors: a single center case series. Cardio-Oncol 6:11 10.1186/s40959-020-00065-9 PMC7388471 32742722

[8] Jang A, Seol YM (2021) Heart rate variability as a non-invasive objective parameter for predicting the occurrence of chemotherapy-induced peripheral neuropathy in patients with gastrointestinal cancer. Anticancer Res 41(5):2637–2645 33952494 10.21873/anticanres.15044

[9] Marstrand SD et al (2021) Vibration perception threshold and heart rate variability as methods to assess chemotherapy-induced neuropathy in women with breast cancer - a pilot study. Cancer Treatment and Research Communications 28:100426 34186438 10.1016/j.ctarc.2021.100426

[10] Li S et al (2020) Transcutaneous auricular vagus nerve stimulation at 20 Hz improves depression-like behaviors and down-regulates the hyperactivity of HPA axis in chronic unpredictable mild stress model rats. Front Neurosci 14:680 32765210 10.3389/fnins.2020.00680 PMC7378324

[11] Chavan SS, Pavlov VA, Tracey KJ (2017) Mechanisms and therapeutic relevance of neuro-immune communication. Immunity 46(6):927–942 28636960 10.1016/j.immuni.2017.06.008 PMC5578398

[12] Mao Y et al (2022) Effects of sub-threshold transcutaneous auricular vagus nerve stimulation on cingulate cortex and insula resting-state functional connectivity. Front Hum Neurosci 16:862443 35496068 10.3389/fnhum.2022.862443 PMC9048677

[13] Garcia-Gonzalez D et al (2023) Biological mechanisms of cancer-related fatigue in breast cancer survivors after treatment: a scoping review. J Cancer Surviv. 10.1007/s11764-023-01477-z 37930591 10.1007/s11764-023-01477-z

[14] Shaffer F, Ginsberg JP (2017) An overview of heart rate variability metrics and norms. Front Public Health 5:258 29034226 10.3389/fpubh.2017.00258 PMC5624990

[15] Gilchrist SC et al (2019) Cardio-oncology rehabilitation to manage cardiovascular outcomes in cancer patients and survivors: a scientific statement from the American Heart Association. Circulation 139(21):e997–e1012 30955352 10.1161/CIR.0000000000000679 PMC7603804

[16] Arslan D, Cevik IU (2022) Interactions between the painful disorders and the autonomic nervous system. Agri 34(3):155–165 35792695 10.14744/agri.2021.43078

[17] Higgins JPT, Chandler LTJ, Tovey D, Churchill R (2016) Methodological Expectations of Cochrane Intervention Reviews. Cochrane

[18] Page MJ et al (2021) PRISMA 2020 explanation and elaboration: updated guidance and exemplars for reporting systematic reviews. BMJ 372:n160 33781993 10.1136/bmj.n160 PMC8005925

[19] McGowan J et al (2016) PRESS peer review of electronic search strategies: 2015 guideline statement. J Clin Epidemiol 75:40–46 27005575 10.1016/j.jclinepi.2016.01.021

[20] Covidence systematic review software. Veritas Health Innovation: Melbourne, Australia.

[21] Team TE (2013) EndNote. Clarivate: Philadelphia, PA.

[22] McHugh ML (2012) Interrater reliability: the kappa statistic. Biochem Med (Zagreb) 22(3):276–282 23092060 PMC3900052

[23] Pace R et al (2012) Testing the reliability and efficiency of the pilot mixed methods appraisal tool (MMAT) for systematic mixed studies review. Int J Nurs Stud 49(1):47–53 21835406 10.1016/j.ijnurstu.2011.07.002

[24] Schünemann HJ, H.J., Vist GE, Glasziou P, Akl EA, Skoetz N, Guyatt GH, *Chapter 14: Completing ‘Summary of findings’ tables and grading the certainty of the evidence*, in *Cochrane Handbook for Systematic Reviews of Interventions version 6.5*, T.J. Higgins JPT, Chandler J, Cumpston M, Li T, Page MJ, Welch VA Editor. 2023, Cochrane: www.training.cochrane.org/handbook.

[25] Tsai HY et al (2022) Predictors of cancer-related fatigue in women with breast cancer undergoing 21 days of a cyclic chemotherapy. Worldviews on Evidence-Based Nursing 19(3):211–218 35229973 10.1111/wvn.12573

[26] FernÁNdez-Lao C et al (2012) Attitudes towards massage modify effects of manual therapy in breast cancer survivors: a randomised clinical trial with crossover design. Eur J Cancer Care 21(2):233–241 10.1111/j.1365-2354.2011.01306.x 22060159

[27] Chen S-C et al (2020) Music, heart rate variability, and symptom clusters: a comparative study. Support Care Cancer 28(1):351–360 31049671 10.1007/s00520-019-04817-x

[28] Aoki M et al (2023) Autonomic function measurements for evaluating fatigue and quality of life in patients with breast cancer undergoing radiation therapy: a prospective longitudinal study. Radiat Oncol 18(1):171 37858146 10.1186/s13014-023-02362-w PMC10585884

[29] Uchida S, Kadoi Y, Saito S (2017) Differences in heart rate variability may be related to the appearance of postoperative pain in patients undergoing breast cancer surgery. JA Clinical Reports 3(1):56 29457100 10.1186/s40981-017-0123-4 PMC5804653

[30] Fagundes CP et al (2011) Sympathetic and parasympathetic activity in cancer-related fatigue: more evidence for a physiological substrate in cancer survivors. Psychoneuroendocrinology 36(8):1137–1147 21388744 10.1016/j.psyneuen.2011.02.005 PMC3128662

[31] Yesil H et al (2018) Is neuropathic pain associated with cardiac sympathovagal activity changes in patients with breast cancer? Neurol Res 40(4):297–302 29447081 10.1080/01616412.2018.1438225

[32] Guimond A-J, Ivers H, Savard J (2019) Is emotion regulation associated with cancer-related psychological symptoms? Psychol Health 34(1):44–63 30516396 10.1080/08870446.2018.1514462

[33] Uchida S, Kadoi Y, Saito S (2017) Effect of low dose remifentanil on postoperative pain relief and heart rate variability in post-anaesthesia care unit. Turk J Anaesthesiol Reanim 45(5):297–302 29114415 10.5152/TJAR.2017.34341 PMC5656165

[34] Chuang C-Y et al (2010) Effects of music therapy on subjective sensations and heart rate variability in treated cancer survivors: a pilot study. Complement Ther Med 18(5):224–226 21056846 10.1016/j.ctim.2010.08.003

[35] Masel EK et al (2016) Heart rate variability during treatment of breakthrough pain in patients with advanced cancer: a pilot study. J Pain Res 9:1215–1220 28003771 10.2147/JPR.S120343 PMC5161332

[36] Lee Y-H et al (2023) Heart rate variability as an indicator of the beneficial effects of Qigong and mindfulness training on the mind–body well-being of cancer survivors. Support Care Cancer 31(1):59 10.1007/s00520-022-07476-7 PMC9761690 36534354

[37] Niederer D et al (2013) Exercise effects on HRV in cancer patients. Int J Sports Med 34(1):68–73 22895874 10.1055/s-0032-1314816

[38] Fournié C et al (2022) Rehabilitation program combining physical exercise and heart rate variability biofeedback in hematologic patients: a feasibility study. Support Care Cancer 30(3):2009–2016 34636946 10.1007/s00520-021-06601-2 PMC8794932

[39] Murofushi KN et al (2023) Preliminary study on establishing a heart rate variability-based method for objectively evaluating bone metastasis pain. In Vivo 37(2):940–947 36881096 10.21873/invivo.13166 PMC10026667

[40] Werthmann PG et al (2025) Efficacy and safety of massage for postoperative stress in colorectal cancer patients: a randomized, controlled, three-arm trial. Front Oncol 15:1439420 39980553 10.3389/fonc.2025.1439420 PMC11840018

[41] Riesenberg H, Lübbe AS (2010) In-patient rehabilitation of lung cancer patients—a prospective study. Support Care Cancer 18(7):877–882 19714371 10.1007/s00520-009-0727-y

[42] Lee Y-H et al (2018) Promoting physical and psychological rehabilitation activities and evaluating potential links among cancer-related fatigue, fear of recurrence, quality of life, and physiological indicators in cancer survivors. Integr Cancer Ther 17(4):1183–1194 30354701 10.1177/1534735418805149 PMC6247550

[43] Cheng T-C et al (2020) The health promoting mindfulness or Qigong educational programs for beneficial lifestyle changes of cancer survivors. J Cancer Educ 35:743–750 31001740 10.1007/s13187-019-01522-5

[44] Fujisawa H, Yamamura K (2024) The impact of animal-assisted therapy on changes in autonomic nervous activity in terminal cancer patients. Int Med J 31(6):169–173

[45] Burch JB et al (2020) Symptom management among cancer survivors: randomized pilot intervention trial of heart rate variability biofeedback. Appl Psychophysiol Biofeedback 45:99–108 32358782 10.1007/s10484-020-09462-3

[46] Hohneck A et al (2025) Differential effects of sound interventions tuned to 432 Hz or 443 Hz on cardiovascular parameters in cancer patients: a randomized cross-over trial. BMC Complement Med Ther 25(1):18 39844155 10.1186/s12906-025-04758-5 PMC11755923

[47] Chai J et al (2024) Effects of the CALM intervention on cancer-related fatigue and heart rate variability in NSCLC: a randomized trial. Future Oncol 20(39):3289–3300 39548708 10.1080/14796694.2024.2428586 PMC11633391

[48] Sammito S, Böckelmann T (2016) Factors influencing heart rate variability. ICF Journal 6:18–22

[49] Kuss O et al (2008) Time domain parameters can be estimated with less statistical error than frequency domain parameters in the analysis of heart rate variability. J Electrocardiol 41(4):287–291 18367200 10.1016/j.jelectrocard.2008.02.014

[50] Okuyama T et al (2000) Development and validation of the cancer fatigue scale: a brief, three-dimensional, self-rating scale for assessment of fatigue in cancer patients. J Pain Symptom Manage 19(1):5–14 10687321 10.1016/s0885-3924(99)00138-4

[51] Shahid A et al (2011) Multidimensional fatigue inventory (MFI). STOP, THAT and one hundred other sleep Scales. Springer, pp 241–243

[52] Stein KD et al (2004) Further validation of the multidimensional fatigue symptom inventory-short form. J Pain Symptom Manage 27(1):14–23 14711465 10.1016/j.jpainsymman.2003.06.003 PMC2547485

[53] Niederer D et al (2015) Heart rate recovery and aerobic endurance capacity in cancer survivors: interdependence and exercise-induced improvements. Support Care Cancer 23(12):3513–3520 25832896 10.1007/s00520-015-2719-4

[54] Jang A, Kim DU, American Society of Clinical Oncology (2020) The relationship between chemotherapy-induced peripheral neuropathy and heart rate variability. J Clin Oncol. 10.1200/JCO.2020.38.4_suppl.82033108243

