# Supplementary material for: Prognostic and diagnostic utility of heart rate variability to predict and understand change in cancer and chemotherapy related fatigue, pain, and neuropathic symptoms: a systematic review
Source: Support Care Cancer. 2025 Nov 11;33(12):1040. doi: 10.1007/s00520-025-10164-x (PMC12602565; doi:10.1007/s00520-025-10164-x)
Supplement: Supplementary file 2 — (PDF 64.4 KB) [file 520_2025_10164_MOESM2_ESM.pdf]

MIXED METHODS APPRAISAL TOOL Non-Randomized Trials

ITEM	S1	S2	1	2	3	4	5
Tsai H, 2021	Yes	Yes	Yes	Yes	Yes	No	Yes
Riesenberg H, 2009	Yes	Yes	Yes	Yes	Yes	Can't tell	Yes
Chuang C, 2010	Yes	Yes	No	No	Yes	Can't tell	Yes
Aoki M, 2023	Yes	Yes	Yes	Yes	No	No	Yes
MaselL E, 2016	Yes	Yes	Yes	Yes	No	No	Yes
Lee Y, 2022	Yes	Yes	No	Yes	Can't tell	Can't tell	Yes
Uchida S, 2017	Yes	YES	Yes	Yes	Yes	No	Yes
Fagundes C, 2011	Yes	Yes	Yes	Yes	No	Yes	Yes
Niederer D, 2013	Yes	Yes	Yes	Yes	No	Yes	Yes
Yesil H, 2018	Yes	Yes	Yes	Yes	Yes	no	Yes
Fournié C, 2021	Yes	Yes	Yes	Yes	No	No	Yes
Murofuashi K, 2023	Yes	Maybe	Yes	Yes	No	Yes	Yes
Guimond A, 2018/2019	Yes	Yes	Yes	Yes	Yes	Yes	Yes
Uchida S, 2017	Yes	Yes	Yes	Yes	Yes	No	Yes
Chou 2024	Yes	Yes	Yes	Yes	No	No	No

MIXED METHODS APPRAISAL TOOL Randomized Trials

ITEM	**S1**	**S2**	**1**	**2**	**3**	**4**	**5**
Lee Y, 2018	Yes	Yes	Can't tell	Yes	No	Can't tell	Yes
Burch J, 2020	Yes	Yes	Yes	Yes	No	Cant tell	No
Cheng 2019	Yes	Yes	Can't tell	Yes	Yes	Can't tell	Yes
Fernandez-Lao 2012	Yes	Yes	Yes	Yes	Cant tell	Yes	Yes
Chen S, 2019	Yes	Yes	Yes	Yes	Yes	Cant tell	Yes
Chai 2024	Yes	Yes	Yes	Yes	No	Yes	Yes
Werthmann 2025	Yes	Yes	Yes	Yes	No	No	Yes
Hohneck 2025	Yes	Yes	Can't Tell	Yes	Yes	No	Yes
